# Transcriptomic Analysis Reveals an NRF2-Mediated Redox and Metabolic Reprogramming in Sorafenib-Resistant Hepatocellular Carcinoma Cells

**DOI:** 10.3390/biotech15010018

**Published:** 2026-02-11

**Authors:** Angelo Michilli, Cristian Bassi, Farzaneh Moshiri, Bruno De Siena, Rosaria Marinaro, Elisa Callegari, Massimo Negrini, Silvia Sabbioni

**Affiliations:** 1 Department of Life Sciences and Biotechnology, University of Ferrara, 44121 Ferrara, Italy; angelo.michilli@unife.it (A.M.); bruno.desiena@unife.it (B.D.S.); 2 Department of Translational Medicine, University of Ferrara, 44121 Ferrara, Italy; cristian.bassi@unife.it (C.B.); mshfzn@unife.it (F.M.); elisa.callegari@unife.it (E.C.); massimo.negrini@unife.it (M.N.); 3 Laboratorio per le Tecnologie delle Terapie Avanzate (LTTA), University of Ferrara, 44121 Ferrara, Italy; 4 Center for Molecular Biology (CDBM), Ferrara University Hospital, 44124 Ferrara, Italy; rosaria.marinaro@edu.unife.it

**Keywords:** hepatocellular carcinoma, sorafenib resistance, NRF2, redox metabolism, oxidative stress, transcriptomics

## Abstract

Despite the advent of immune checkpoint inhibitor-based regimens, sorafenib remains an important therapeutic option for patients with advanced hepatocellular carcinoma (HCC) who are ineligible for immunotherapy. However, its clinical efficacy is limited by the emergence of drug resistance, whose underlying molecular mechanisms remain incompletely understood. To investigate these mechanisms, we established a murine model of acquired sorafenib resistance and performed comparative RNA sequencing of sorafenib-sensitive versus -resistant Hep55.1C hepatoma cells. Transcriptomic profiling revealed a distinct resistance-associated signature comprising 1264 significantly deregulated genes (adjusted *p* < 0.03, fold change > 3.0). Pathway analysis and Gene Set Enrichment Analyses (GSEA) indicated a coordinated downregulation of metabolic and intercellular signaling pathways, accompanied by marked upregulation of redox-regulatory, mitochondrial and cellular stress-response programs. Genes transcriptionally regulated by nuclear factor erythroid 2-related factor 2 (NRF2) including Gpx4, Txn1, Txnrd1, Hmox1, Fth1, Taldo1, Phgdh, and MafG, involved in antioxidant defense, ferroptosis suppression and metabolic rewiring, were all upregulated in resistant cells. Pharmacological inhibition of NRF2 activity using brusatol restored sensitivity to sorafenib, functionally implicating NRF2-dependent pathways in the maintenance of the resistant phenotype. These findings demonstrate that acquired sorafenib resistance in HCC is associated with a stable NRF2-driven transcriptional and metabolic reprogramming that enhances antioxidant capacity, suppresses ferroptosis and promotes tumor cell survival. Targeting NRF2-regulated redox metabolism may therefore represent a promising strategy to overcome therapeutic resistance in HCC.

## 1. Introduction

Hepatocellular carcinoma (HCC) represents the most prevalent primary liver malignancy, accounting for approximately 85% of all primary liver cancers [1] and ranking among the leading causes of cancer-related mortality worldwide [2]. Despite advances in surveillance and therapeutic strategies, the prognosis of advanced HCC remains poor, largely due to late diagnosis and the limited efficacy of systemic treatments.

For more than a decade, sorafenib served as the first-line systemic therapy for patients with advanced, unresectable HCC. Sorafenib is a multikinase inhibitor targeting both tumor-intrinsic and tumor-extrinsic pathways, including RAF kinases, vascular endothelial growth factor receptors (VEGFR1–3), platelet-derived growth factor receptor β (PDGFR-β), and c-KIT [3]. In addition to its anti-proliferative and anti-angiogenic activities, sorafenib perturbs mitochondrial function and promotes oxidative stress, contributing to its cytotoxic effects [4].

In recent years, the therapeutic landscape of advanced HCC has evolved with the introduction of additional multikinase inhibitors [5] and immune checkpoint inhibitor-based therapies. The combination of atezolizumab and bevacizumab has demonstrated superior efficacy compared to sorafenib and is currently the preferred first-line option for eligible patients [6]. Nevertheless, approximately 70% of patients either fail to respond or develop early disease progression, including hyperprogressive disease [7,8,9]. Hence, a significant fraction of patients remains ineligible for immunotherapy, thus maintaining a relevant clinical role for sorafenib.

A major limitation of sorafenib therapy is the development of intrinsic or acquired resistance. Unlike resistance to targeted therapies driven by recurrent genetic alterations, sorafenib resistance has been associated with a broad spectrum of molecular and cellular adaptations rather than specific driver mutations. These adaptive responses include metabolic rewiring, activation of survival signaling pathways, modulation of oxidative stress responses and evasion of regulated cell death programs [10,11,12,13,14,15,16,17,18,19]. However, the relative contribution and integration of these mechanisms remain incompletely understood, limiting their translational exploitation.

Among adaptive stress-response pathways, the nuclear factor erythroid 2-related factor 2 (NRF2) signaling axis has emerged as a central regulator of cellular redox homeostasis and metabolic plasticity. NRF2 orchestrates the transcription of genes involved in antioxidant defense, detoxification, intermediary metabolism and ferroptosis suppression, thereby promoting cell survival under conditions of oxidative or metabolic stress [20,21]. Constitutive or inducible NRF2 activation has been linked to resistance to chemotherapy including cisplatin, doxorubicin, paclitaxel, as well as to radioresistance across multiple tumor types [22,23,24,25].

In this study, we aimed at elucidating the transcriptional programs underlying acquired sorafenib resistance in HCC by performing comparative RNA sequencing of sorafenib-sensitive and -resistant murine hepatoma cells. By integrating differential expression analysis, pathway enrichment detection and functional pharmacological inhibition, we identify NRF2-dependent redox and metabolic reprogramming as a prominent and functionally relevant feature of the resistant phenotype. Our findings provide a coherent framework linking oxidative stress adaptation, ferroptosis suppression and metabolic flexibility to sorafenib resistance and highlight NRF2-regulated pathways as potential therapeutic vulnerabilities.

## 2. Materials and Methods

### 2.1. Cell Culture and Generation of Sorafenib-Resistant Cells

Murine hepatoma Hep55.1C and Hep53.4 cell lines, originally derived from C57BL/6 mice, were obtained from CLS Cell Lines Service GmbH (Heidelberg, Germany). Cells were maintained in Dulbecco’s Modified Eagle Medium (DMEM; Gibco, brand of Thermo Fisher Scientific, Monza, Italy) supplemented with 10% fetal bovine serum (FBS; Sigma-Aldrich, brand of Merck Life Sciences, Milano, Italy), 1% penicillin/streptomycin and 2 mM L-glutamine at 37 °C in a humidified atmosphere containing 5% CO_2_. Sorafenib-resistant Hep55.1C cells were generated through an in vivo selection strategy as previously described the cells were established in a published previous study [26]. Briefly, C57BL/6J mice bearing Hep55.1C-derived tumors were treated with sorafenib. While most tumors initially responded, regrowth was observed in a subset after approximately one month of treatment. Tumors exhibiting regrowth were explanted, and derived cells were cultured in vitro in the presence of sorafenib (10 µM). Surviving cell populations were expanded and continuously maintained in sorafenib-containing medium to stabilize the resistant phenotype (Hep55.1C-RES or more briefly H55-RES). Resistance was validated by cell viability assays demonstrating sustained proliferation under sorafenib exposure compared with parental cells, which served as sensitive controls.

### 2.2. RNA Extraction, Library Preparation, and Sequencing

Total RNA was isolated using the Maxwell RSC simplyRNA Cells Kit (Promega Italia, Milano, Italy) on the Maxwell RSC automated platform, following the manufacturer’s instructions. RNA integrity was assessed using an Agilent 2100 Bioanalyzer, and only samples with RNA integrity numbers (RIN) greater than 7.0 were used for downstream analyses. Ribosomal RNA depletion was performed using the Qiagen QIAseq FastSelect RNA Removal Kit, followed by library preparation with the Qiagen QIAseq Stranded Total RNA Library Kit (Qiagen, Milano, Italy). This workflow included RNA fragmentation, reverse transcription, second-strand synthesis, end repair, A-tailing, adapter ligation, and library amplification. Sequencing was carried out on an Illumina NextSeq 500 platform using the NextSeq High Output Kit v2 (150 cycles), generating an average of approximately 40 million paired-end reads per sample.

### 2.3. Bioinformatic Processing and Differential Expression Analysis

Raw sequencing reads were subjected to quality control using FastQC (v0.11.5) [Available online at: http://www.bioinformatics.babraham.ac.uk/projects/fastqc]. (accessed on 11 December 2017). High-quality reads were aligned to the mouse reference genome (GRCm38) using HISAT2 [27] with a pre-built genome index. Transcript assembly and quantification were performed using StringTie [28]. Raw count matrices were imported into R and normalized using the DESeq2 Bioconductor package [28,29]. Differential gene expression analysis was conducted with DESeq2 (v1.42.0, R v4.3.1). Genes with an adjusted *p*-value < 0.03 (Benjamini–Hochberg correction) and an absolute fold change > 3.0 were considered significantly differentially expressed.

### 2.4. Functional Enrichment and Pathway Analysis

Functional annotation of differentially expressed genes (DEGs) was performed using the Kyoto Encyclopedia of Genes and Genomes (KEGG) database. Enrichment analysis was conducted via the KOBAS-i (KOBAS 3.0) online web platform (http://kobas.cbi.pku.edu.cn/) (accessed on 27 February 2024). [30], applying a false discovery rate (FDR) threshold of 0.05. Pathways were grouped into functional macro-categories according to KEGG hierarchy and biological relevance. For each list, an over-representation analysis (ORA) was performed to identify biological pathways significantly enriched among the genes. Only the top five significantly enriched pathways were displayed per cluster.

### 2.5. Gene Set Enrichment Analysis (GSEA)

To complement the discrete differential expression approach and identify coordinated transcriptional programs, Gene Set Enrichment Analysis (GSEA) was performed using the GSEA Desktop Application (v4.1.0, Broad Institute) [31]. Ranked gene lists were generated based on the DESeq2 statistic, and enrichment was tested against the MSigDB Hallmark and Reactome gene set collections. Enrichment scores (ES) were calculated using 1000 gene set permutations, and FDR-adjusted *q*-values < 0.25 were considered significant, consistent with GSEA standards. Normalized enrichment scores (NES) and leading-edge subsets were extracted to identify dominant biological signatures. The results were visualized using ClusterProfiler (v4.12.6) [32].

### 2.6. Identification of NRF2-Dependent Transcriptional Programs

NRF2 target genes were annotated according to the curated list described by Hayes & Dinkova-Kostova [33] and cross-referenced with the MSigDB gene set “HALLMARK_OXIDATIVE_STRESS_RESPONSE.” Overlaps between DEGs and known NRF2 targets were quantified using Fisher’s exact test, and expression levels were visualized as fold change heatmaps. Representative NRF2-regulated genes (e.g., *Gpx4*, *Txn1*, *Txnrd1*, *Hmox1*, *Fth1*, *Taldo1*, and *Phgdh*) were validated for upregulation in resistant cells and confirmed to belong to the NRF2-ARE regulatory network.

### 2.7. Drugs

For in vitro experiments, brusatol (Cat. No. HY-19543) and sorafenib (Bay 43-9006; Cat. No. HY-10201) were purchased from MedChemExpress (Monmouth Junction, NJ, USA). Both compounds were dissolved in dimethyl sulfoxide (DMSO).

### 2.8. Cell Viability Assay

Cell viability in vitro was assessed using the PrestoBlue™ HS Cell Viability Reagent (#P50200; Invitrogen, Thermo Fisher Scientific, Carlsbad, CA, USA) in accordance with the manufacturer’s instructions. All assays were performed in triplicate and quantified using a Tecan Infinite 200 Pro M Plex microplate reader (Tecan, Grodig, Austria GmbH). Drug efficacy was evaluated by determining the half-maximal inhibitory concentration (IC_50_).

### 2.9. Quantitative Droplet Digital PCR (ddPCR) Analysis

Total RNA was isolated as described above and reverse-transcribed into cDNA using SuperScript™ IV Reverse Transcriptase (Thermo Fisher Scientific) at 42 °C for 1 h, according to manufacturer’s instructions. Droplet digital PCR was performed using a QX200 ddPCR system (Bio-Rad Italia, Segrate, MI, Italy). Briefly, reactions (20 µL final volume) were prepared using QX200™ ddPCR™ EvaGreen Supermix (Bio-Rad), gene-specific primers and cDNA template. The genes analyzed included mouse *Nrf2*, *Gpx4*, *Txn1*, *Txnrd1*, *Hmox1*, *Fth1*, *Taldo1*, *Phgdh* and *β-actin* (Table 1). Droplets were generated with the QX200 Droplet Generator (Bio-Rad) and PCR amplification was carried out on a thermal cycler using the following cycling conditions: denaturation 94 °C/30 s, annealing 56 °C/30 s, extension 72 °C/30 s, for 35 cycles. After amplification, droplets were read on a QX200 Droplet Reader (Bio-Rad). Data were analyzed with QuantaSoft software (Bio-Rad) (version 1.7) by setting fluorescence thresholds to discriminate positive and negative droplets. Absolute target concentrations were reported as copies per µL of reaction and, where indicated, normalized to mouse β-actin levels, used as a reference gene. All samples were run in technical duplicates and included no-template controls and no-reverse transcriptase controls.

### 2.10. Western Blotting and Antibodies

Cells were harvested and lysed in radioimmunoprecipitation assay (RIPA) buffer (#R0278; Sigma–Aldrich, St. Louis, MO, USA) as previously described [26]. Equal amounts of protein (10 μg per sample) were resolved by SDS–PAGE using 4–15% Tris–glycine gels (no. 4561083, Bio-Rad) and subsequently transferred onto PVDF membranes (no. 1704156, Bio-Rad). After blocking with a 5% blocking agent, membranes were incubated overnight at 4 °C with the following rabbit polyclonal antibodies: HO-1 (E3F4S, #43966, Cell Signaling Technology, Danvers, MA, USA) and NRF2 (NBP1-32822, Novus Biologicals, Centennial, CO, USA). A mouse monoclonal anti-glyceraldehyde-3-phosphate dehydrogenase (GAPDH) antibody (clone 2D9, TA802519; OriGene Technologies, Rockville, MD, USA) was used as a loading control. For chemiluminescent detection, membranes were incubated with a horseradish peroxidase–conjugated secondary antibody (#7074; Cell Signaling Technology), and signals were visualized using Clarity Western ECL Blotting Substrate (#170-5060; Bio-Rad). Images were acquired with a ChemiDoc imaging system (Bio-Rad), and band intensities were quantified using ImageJ software (https://imagej.nih.gov) (version 1.54). Protein expression levels were normalized to GAPDH.

### 2.11. Statistical Analysis

Statistical analyses of RNA-seq data were performed in R (v4.4.1) using the DESeq2 package (v1.42.0). Differential gene expression was assessed by modeling count data with a negative binomial distribution and applying Wald tests, with *p* values adjusted for multiple testing using the Benjamini–Hochberg method. Genes with an adjusted *p* value < 0.03 and an absolute fold change > 3 were considered significantly differentially expressed. Volcano plots, heatmaps, and bar graphs were generated to visualize differential gene expression and pathway enrichment results. Statistical analyses of ddPCR data were performed using GraphPad Prism 6.0 (GraphPad Software, La Jolla, CA, USA). Comparisons between groups were carried out using an unpaired Student’s *t*-test when variances were equal, as determined by an F-test. A *p* value ≤ 0.05 was considered statistically significant. Where appropriate, data are presented as mean ± standard deviation (SD).

## 3. Results

### 3.1. Transcriptomic Profiling Identifies a Distinct Gene Expression Signature Associated with Sorafenib Resistance

To investigate the molecular basis of acquired sorafenib resistance, we performed a comparative transcriptomic analysis of sorafenib-sensitive and -resistant murine hepatocellular carcinoma Hep55.1C cells. Sorafenib-resistant cells were previously established by the authors and reported in a published previous study and the approach employed for the development is briefly described in Section 2 [26].

Genome-wide RNA sequencing was conducted on a total of eight biological samples, including two sorafenib-sensitive parental controls and six independently derived sorafenib-resistant Hep55.1C sublines.

**Figure 1:**
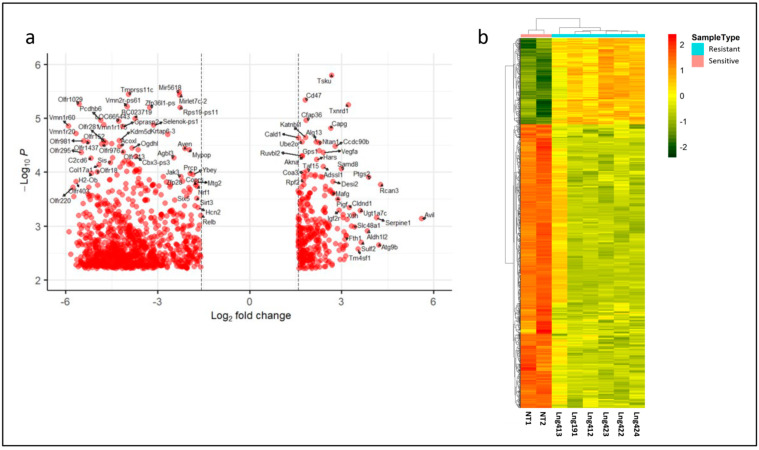
*Differential gene expression and hierarchical clustering in sorafenib-resistant versus -sensitive Hep55.1C cells.* (**a**) Volcano plot illustrating the global transcriptional differences between sorafenib-resistant and -sensitive Hep55.1C cells. The *x*-axis represents the log_2_ fold change, indicating the magnitude and direction of differential expression, while the *y*-axis shows the −log_10_ (*p*-value), reflecting statistical significance. Upregulated genes (*n* = 294) in resistant cells are displayed on the right side of the plot, whereas downregulated genes (*n* = 970) are on the left. For clarity reasons, only part of the dysregulated gene names is shown. (**b**) Unsupervised hierarchical clustering of differentially expressed genes distinguishes resistant from sensitive phenotypes. Each row represents an individual gene, and each column corresponds to a biological replicate. Samples from sorafenib-sensitive Hep55.1C cells are shown in pink, and those from sorafenib-resistant cells in blue. Gene expression values were normalized to the mean expression of each gene across the dataset. The heatmap color scale represents relative expression levels, with red indicating high expression, green indicating low expression, and yellow representing values near the mean. The analysis reveals a clear segregation between resistant and sensitive samples, underscoring a distinct transcriptional landscape associated with the resistant phenotype.

Differential expression analysis identified 1264 genes significantly deregulated between resistant and sensitive cells (adjusted *p* value < 0.03, Benjamini–Hochberg correction; absolute fold change > 3.0). Among these, 970 genes were downregulated and 294 were upregulated in resistant cells (Figure 1a). Unsupervised hierarchical clustering based on the expression of these differentially expressed genes clearly segregated resistant from sensitive samples into two distinct clusters (Figure 1b). This well-defined separation indicates that acquired sorafenib resistance is associated with a stable and reproducible transcriptional reprogramming. The consistency of this signature across multiple independently generated resistant sublines suggests the activation of common adaptive pathways that may contribute functionally to the acquisition of the resistant phenotype.

### 3.2. Pathway Enrichment Analysis Reveals Coordinated Metabolic and Stress-Adaptive Programs in Resistant Cells

To gain insight into the biological processes associated with the observed transcriptional changes, we performed pathway enrichment analysis of differentially expressed genes using the KEGG database via the KOBAS-i platform.

**Figure 2:**
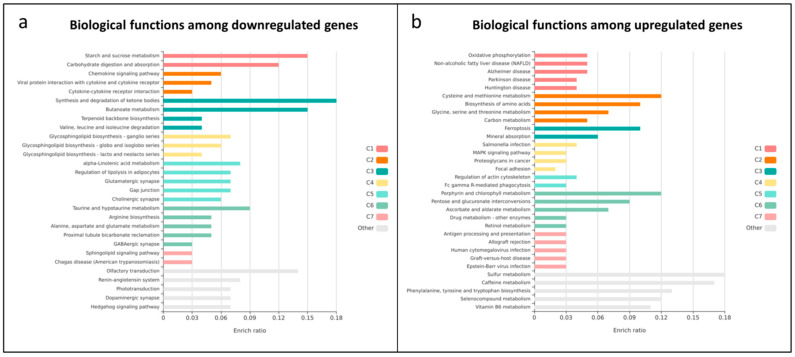
*Functional enrichment analysis of dysregulated genes in sorafenib-resistant Hep55.1C cells.* (**a**) KEGG pathway enrichment of downregulated genes in resistant versus sensitive cells, performed with KOBAS-i. The *x*-axis shows the enrichment ratio and the *y*-axis the enriched pathways. Colored bars indicate functional clusters. The five most significantly enriched pathways are displayed per cluster. Downregulated genes primarily map to pathways involved in energy metabolism, cell signaling, and lipid/amino acid metabolism. (**b**) KEGG enrichment of upregulated genes, using the same analytical workflow. Upregulated genes are enriched in oxidative phosphorylation, amino acid and one-carbon metabolism, and redox/ferroptosis-related pathways, reflecting metabolic and stress-adaptive mechanisms associated with sorafenib resistance.

Genes downregulated in sorafenib-resistant cells were significantly enriched in pathways related to energy metabolism, lipid and amino acid metabolism, and intercellular signaling (Figure 2a). These pathways were grouped into functional macro-categories: (C1) Energy and nutrient metabolism; (C2) Cellular and immune signaling; (C3) Lipid and short-chain amino acid metabolism; (C4) Glycosphingolipid biosynthesis; (C5) Unsaturated fatty acid metabolism and neurotransmission; (C6) Amino acid metabolism and renal function; (C7) Sphingolipid signaling and infection; (Other) Neurosensory and hormonal signaling. These results suggest that sorafenib resistance is associated with a generalized downregulation of pathways related to nutrient metabolism, lipid processing, and intercellular communication, potentially reflecting a metabolic shift toward adaptive survival states.

In contrast, genes upregulated in resistant cells were enriched in pathways associated with redox regulation, mitochondrial function, amino acid and one-carbon metabolism, cellular stress responses and detoxification processes (Figure 2b). Enriched categories included: (C1) Mitochondrial dysfunction and disease; (C2) Amino acid and one-carbon metabolism; (C3) Cellular homeostasis, death, and immunity; (C4) Oncogenic signaling and adhesion; (C5) cytoskeletal remodeling; (C6) Heme, carbohydrate, drug, and vitamin metabolism; (C7) Immune response and viral infection; (Other) Aromatic and seleno compound metabolism. Taken together, these findings suggest that resistant cells undergo extensive metabolic rewiring toward enhanced oxidative metabolism and redox buffering capacity, hallmarks of stress-adapted, therapy-tolerant cancer states.

### 3.3. Gene Set Enrichment Analysis Identifies NRF2-Driven Transcriptional Programs in Sorafenib-Resistant Cells

To complement differential expression analysis and capture coordinated transcriptional programs, we performed Gene Set Enrichment Analysis (GSEA) using ranked gene lists derived from DESeq2 statistics. This approach avoids reliance on arbitrary fold-change thresholds and enables the identification of pathway-level alterations.

**Figure 3:**
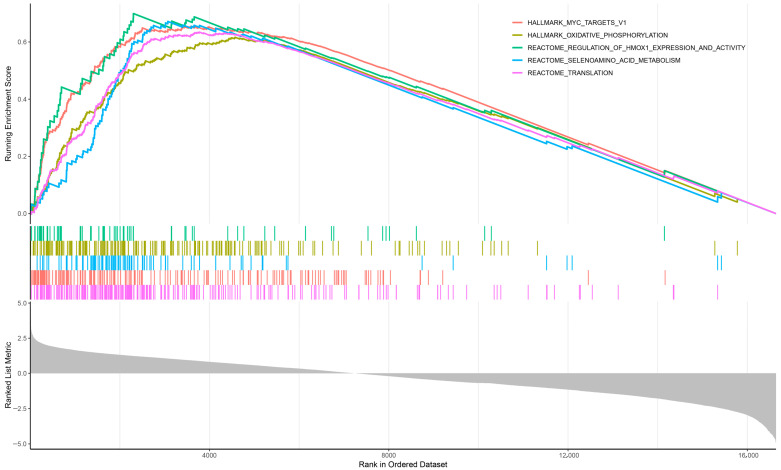
*Gene Set Enrichment Analysis (GSEA).* Representative examples of gene sets significantly enriched of overexpressed genes in sorafenib-resistant cells are shown. Each vertical colored line corresponds to a gene within the indicated gene set, with genes ranked from most overexpressed to most downregulated. As evident from the plots, the majority of genes in these sets display marked overexpression. The gray shaded area shows the Ranked List Metric, corresponding to the distribution of ranking scores for all genes, from the most up-regulated (**left**) to the most down-regulated (**right**). Five gene sets associated with functionally relevant pathways were selected from the comprehensive list provided in Supplementary Table S1.

GSEA revealed a strong and consistent enrichment of gene sets associated with oxidative stress responses, redox homeostasis, mitochondrial metabolism and detoxification among genes overexpressed in sorafenib-resistant cells (Figure 3; Supplementary Table S1). Among these, multiple gene sets corresponded to transcriptional programs regulated by nuclear factor erythroid 2-related factor 2 (NRF2), a master regulator of cellular antioxidant and metabolic responses.

**Figure 4:**
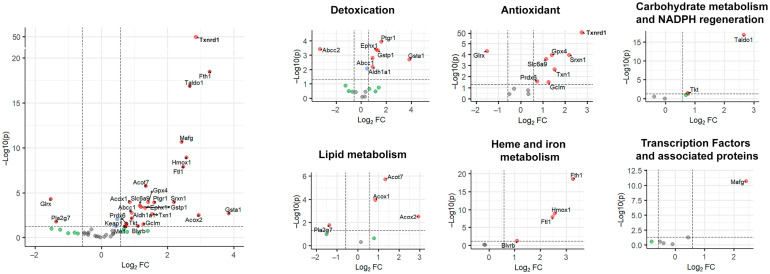
*NRF2 is a key regulator of sorafenib resistance.* From comparison of Hep55.1C sorafenib resistant versus susceptible parental cells, the large panel on the left displays the trend of all known NRF2-responsive genes described by Hayes et al. [33]. The smaller sub-panels highlight genes grouped by major biological functions. For all the diagrams, the *Y*-axis shows statistical significance (−Log10 *p* value) and the *X*-axis indicates the fold change (Log10). Red dots indicate the genes that exhibit a fold change > 3 and a *p* value < 0.03. Green dots indicate the genes with a fold change > 3 but a *p* value > 0.03. Gray dots indicate the genes with a fold change < 3 and a *p* value > 0.03. Genes located in the upper-right quadrant of each panel correspond to the upregulated genes with a fold change > 3 and a *p* value < 0.03. For each gene, the fold change was calculated based on the ratio between the mean expression values of resistant versus sensitive cells, and statistical significance (*p* values adjusted for multiple testing) was assessed using the Benjamini–Hochberg method.

**Figure 5:**
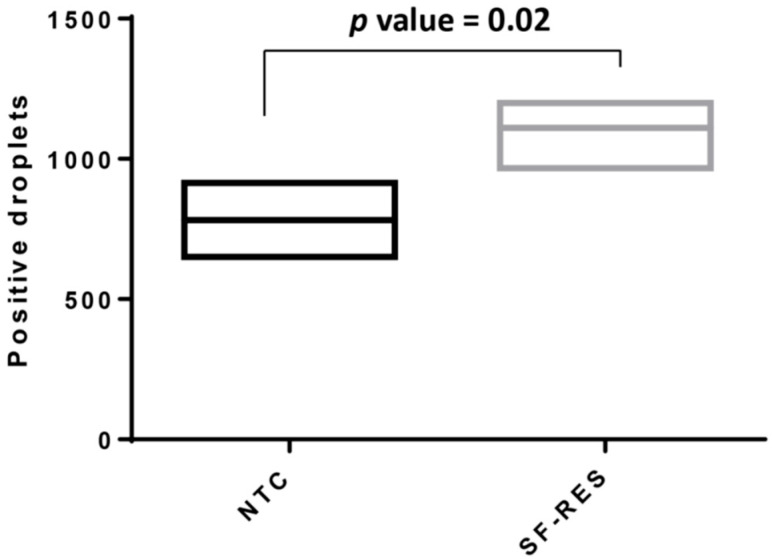
*Expression of Nrf2 gene in parental and resistant cells.* Quantitative analyses were performed by droplet digital PCR. The analysis shows the results from two control parental Hep55.1C cells (NTC) and five independent H55-RES cell lines (SF-RES). There is a significant (*p* value = 0.02) slight upregulation (1.3-fold change) of Nrf2 mRNA in sorafenib-resistant cells H55-RES.

Canonical NRF2 target genes, including *Gpx4*, *Txn1*, *Txnrd1*, *Hmox1*, *Fth1*, *Taldo1*, *Phgdh*, and *MafG*, were all present in enriched gene sets exhibiting overexpression in resistant cells. These genes are involved in glutathione and NADPH metabolism, thioredoxin-dependent redox control, heme detoxification and ferroptosis suppression. The mechanism and the magnitude of fold change in NRF2-induced genes are illustrated in Figure 4. Among genes, it is notable that Nrf2 mRNA itself is upregulated in H55-RES cells (Figure 5) compared to parental Hep55.1C cells.

The coordinated induction of these pathways strongly implicates NRF2-dependent transcriptional programs characterized by enhanced antioxidant capacity and activation of redox-regulatory programs that collectively represent the features of the sorafenib-resistant phenotype.

### 3.4. Sorafenib Rapidly Induces an NRF2-Dependent Transcriptional Response in Sensitive Hepatoma Cells

To determine whether activation of NRF2-regulated pathways represents an early adaptive response to sorafenib exposure, we analyzed the expression of NRF2 target genes shortly after drug treatment in parental sorafenib-sensitive Hep55.1C cells. To assess whether this response was cell-line specific, we performed parallel analyses in an independent murine hepatoma cell line, Hep53.4.

**Figure 6:**
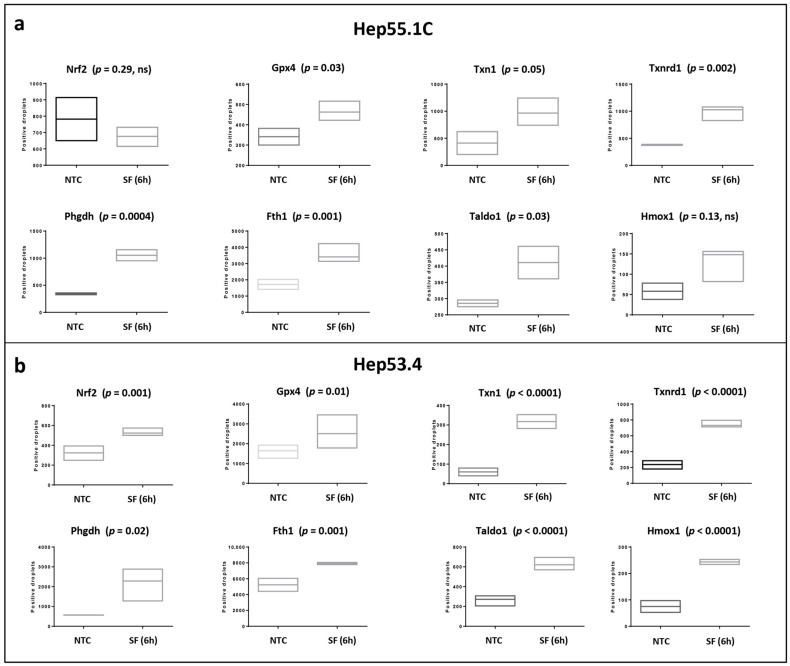
*Induction of NRF2-responsive genes following sorafenib treatment in parental Hep55.1C (**a**) and Hep53.4 (**b**) cells.* Quantitative analyses were performed by droplet digital PCR. Gene expression was analyzed 6 h after sorafenib exposure to minimize potential confounding effects arising from induction of cell death processes. Canonical NRF2 target genes were significantly upregulated at this early time point, indicating that molecular mechanisms contributing to sorafenib resistance are initiated rapidly upon drug exposure. In contrast, *Nrf2* mRNA levels were not increased in Hep55.1C cells, suggesting that NRF2 activation occurs through canonical post-transcriptional KEAP1 control mechanisms.

Gene expression was evaluated 6 h after sorafenib treatment to minimize potential confounding effects related to secondary stress responses or cell death. At this early time point, multiple canonical NRF2 target genes were significantly upregulated in both Hep55.1C and Hep53.4 cells (Figure 6a,b), indicating rapid activation of a NRF2-dependent transcriptional program following sorafenib exposure.

Interestingly, *Nrf2* mRNA level was not significantly increased in Hep55.1C cells at this time point, whereas it was induced in Hep53.4 cells. This suggests that early NRF2 activation in Hep55.1C cells is likely to occur through post-transcriptional mechanisms, consistent with canonical KEAP1-dependent regulation triggered by oxidative stress. Collectively, these findings indicate that NRF2-driven cytoprotective responses are initiated rapidly upon sorafenib treatment and become stable in the early establishment of drug tolerance.

### 3.5. Pharmacological Inhibition of NRF2-Dependent Protein Accumulation Restores Sorafenib Sensitivity

To further support the involvement of NRF2-dependent pathways to the maintenance of sorafenib resistance, we treated sorafenib-resistant Hep55-RES cells with brusatol, a compound known to suppress NRF2 signaling primarily through inhibition of protein synthesis rather than direct transcriptional repression [34,35,36].

**Figure 7:**
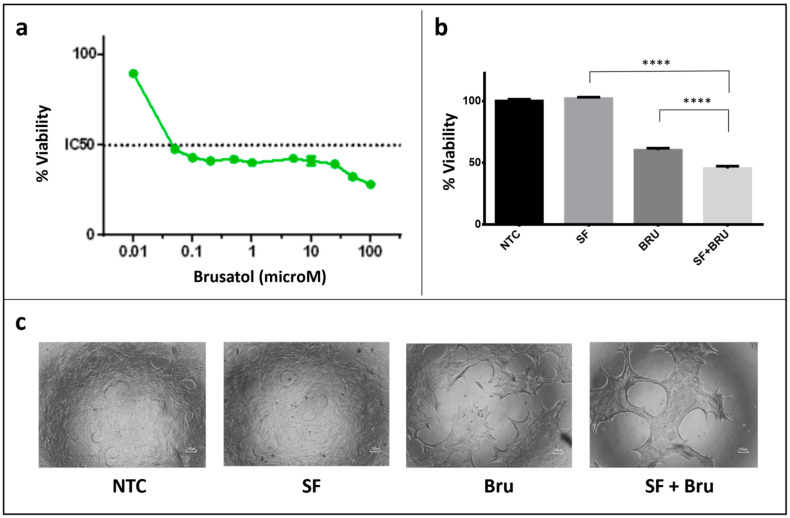
*Brusatol-mediated inhibition of NRF2 restores sensitivity to sorafenib.* (**a**) Hep55-RES cells remain fully viable following treatment with 7.5 μM sorafenib. In contrast, co-treatment with the NRF2 inhibitor brusatol induces a marked reduction in cell viability within 24 h, even at concentrations below 0.1 μM. (**b**) Quantification of cell viability in Hep55-RES cells after 24 h of treatment with sorafenib and/or brusatol shows that the strongest decrease is caused by the combined treatment (SF + Bru), compared with sorafenib or brusatol alone (**** corresponds to *p* value < 0.0001). SF, sorafenib (7.5 μM); Bru, brusatol (0.1 μM). (**c**) Representative micrographs of Hep55-RES cells 24 h after the initiation of brusatol treatment. Brusatol alone and in combination with sorafenib visibly reduce cell viability compared with control conditions (NTC and sorafenib alone). Scale bar = 100 µm.

As expected, Hep55-RES cells remained fully viable upon treatment with sorafenib alone (7.5 μM), confirming the stability of the resistant phenotype (Figure 7). In contrast, co-treatment with brusatol resulted in a pronounced reduction in cell viability within 24 h, even at low brusatol concentrations (Figure 7). This effect was significantly greater than that observed with either agent alone, indicating a strong sensitizing interaction.

**Figure 8:**
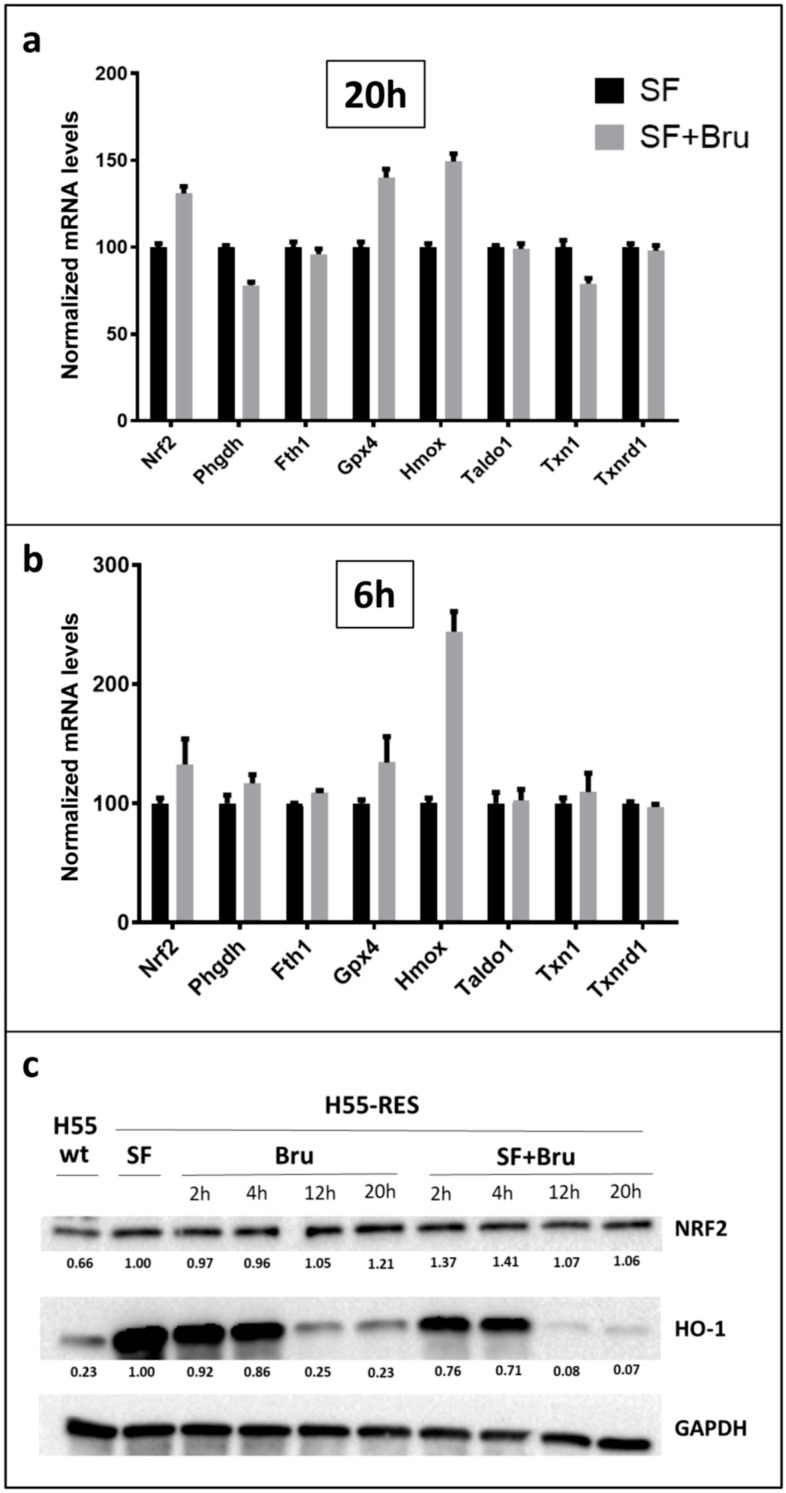
*RNA and protein expression of NRF2 and NRF2-inducible genes following brusatol treatment in H55-RES cells.* (**a**) Quantitative analysis of mRNA levels of selected NRF2 target genes were performed by droplet digital PCR. 20 h after initiation of brusatol treatment (0.1 μM), expressed relative to mRNA levels measured at the same time point in sorafenib-treated resistant H55-RES cells (7.5 μM). (**b**) The same analysis performed 6 h after initiation of brusatol treatment. (**c**) Time-course analysis of protein expression at 2, 4, 12, and 20 h following brusatol treatment. Notably, heme oxygenase 1 (HO-1, encoded by Hmox1) exhibits an expression pattern opposite to that observed at the mRNA level, showing a marked reduction in protein abundance, particularly at 12 and 20 h, when cell viability is strongly reduced. HO-1 protein levels are more profoundly decreased upon combined treatment with sorafenib and brusatol, correlating with the pronounced loss of cell viability observed under these conditions.

Despite the marked reduction in cell viability observed at approximately 24 h after treatment initiation, transcriptional analysis at the same time point revealed a heterogeneous response of NRF2 target genes following brusatol treatment (Figure 8a). While some transcripts, such as *Phgdh* and *Txn1*, showed partial downregulation, others, including *Fth1*, *Taldo1*, and *Txnrd1*, were unchanged. Conversely, *Nrf2*, *Gpx4*, and *Hmox1* mRNA levels were upregulated (Figure 8a). Notably, the induction of NRF2-target genes was even more pronounced at earlier time points and generally declined by 20 h (Figure 8a,b), coinciding with the onset of a marked reduction in cell viability. This apparent discordance between mRNA expression and cell viability prompted further analysis at the protein level.

Immunoblotting demonstrated a rapid and substantial decrease in HO-1 protein levels following brusatol exposure, particularly under combined sorafenib–brusatol treatment (Figure 8c). Protein reduction was detectable as early as 2 h after treatment initiation and became progressively more pronounced over time, despite sustained or increased transcript levels. These findings are consistent with the known post-transcriptional mechanism of action of brusatol and indicate that loss of antioxidant protein accumulation, rather than transcriptional repression, underlies the observed loss of cell viability.

Together, these results provide functional evidence that NRF2-dependent antioxidant and metabolic programs contribute to the maintenance of sorafenib resistance and that disruption of NRF2-driven protein expression is sufficient to restore drug sensitivity in resistant HCC cells.

## 4. Discussion

In this study, we characterized the transcriptional landscape associated with acquired resistance to sorafenib in hepatocellular carcinoma and identified a coordinated NRF2-dependent redox and metabolic reprogramming as a prominent feature of the resistant phenotype. Our findings indicate that resistance to sorafenib in HCC is primarily driven by transcriptional reprogramming rather than genetic mutations. This is consistent with the published literature [10,11,12,13,14,15,16,17,18,19] and with exome sequencing analyses performed in the different sorafenib-resistant Hep55.1C cell lines, which did not reveal mutations that could be associated with the development of resistance. 

Through integrative transcriptomic analyses and functional pharmacological intervention, our results indicate that adaptation to oxidative stress and reinforcement of antioxidant defenses play a central role in sustaining tumor cell survival under prolonged sorafenib exposure.

Comparative RNA sequencing of sorafenib-sensitive and -resistant Hep55.1C cells revealed a highly reproducible transcriptional signature shared across multiple independently generated resistant sublines. This convergence strongly suggests that resistance emerges through common adaptive programs. In line with previous studies reporting the absence of recurrent genetic alterations driving sorafenib resistance, our findings provide a coherent, logical and novel framework of the numerous mechanisms that collectively can lead to resistance to sorafenib and support the concept that resistance is largely mediated by transcriptional and metabolic plasticity. Pathway enrichment analyses revealed an upregulated pattern involved in redox regulation, mitochondrial function, stress responses and detoxification. This coordinated shift is consistent with the emergence of a stress-tolerant cellular state, in which cellular functions are directed toward survival under sustained pharmacological pressure.

**Figure 9:**
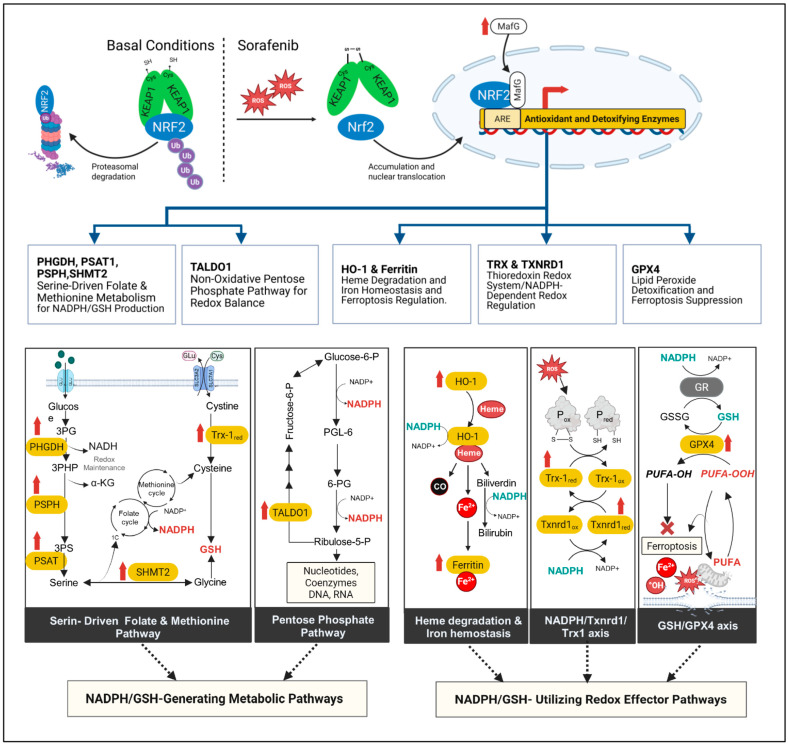
Schemes of mechanisms linking sorafenib resistance to a coordinated NRF2-dependent antioxidant and metabolic detoxification program. The upper panel illustrates the mechanism of NRF2 activation under sorafenib-induced oxidative stress. Under basal conditions (**left**), KEAP1 binds to NRF2 and promotes its ubiquitination, ensuring continuous proteasomal degradation. Upon sorafenib treatment (**center**), increased ROS oxidize critical cysteine residues in KEAP1, disrupting the KEAP1–NRF2 interaction and allowing NRF2 to stabilize, accumulate, and translocate into the nucleus. Once in the nucleus, NRF2 heterodimerizes with MafG—whose expression is concurrently upregulated (red upward arrow)—and binds ARE sequences to activate a cytoprotective transcriptional program (**right**). This response enhances metabolic and antioxidant pathways that support cell survival and contribute to sorafenib resistance in HCC. The lower panels summarize five NRF2-regulated metabolic and antioxidant modules, encompassing both NADPH/GSH-producing pathways and NADPH/GSH-consuming antioxidant systems. The NADPH/GSH-producing pathways include: (i) upregulation of PHGDH, PSAT1, PSPH, and SHMT2, which boosts serine/one-carbon metabolism and increases NADPH and GSH synthesis; the same panel shows that elevated TRX-1, which reduces cystine to cysteine, provides additional substrate for GSH production; (ii) induction of TALDO1, supporting ribose-5-phosphate generation and sustaining NADPH levels through PPP recycling. The NADPH/GSH-consuming antioxidant systems include: (i) Hmox1-driven heme degradation, with ferritin sequestering Fe^2+^ to limit ROS production, and biliverdin reductase linking this module to NADPH consumption; (ii) the NADPH-dependent thioredoxin cycle, which restores oxidized protein thiols under ROS stress; and (iii) GPX4-mediated detoxification of PUFA-OOH to PUFA-OH using GSH, with GSH regenerated by NADPH-dependent glutathione reductase (GR, gray oval), thereby preventing lipid peroxidation and ferroptosis. Red upward arrows indicate gene upregulation, orange round rectangles denote enzymes that are transcriptionally NRF2-induced. Abbreviations: 3PG: 3-phosphoglycerate; 3PHP: 3-phosphohydroxypyruvate; 3PS: 3-phosphoserine; α-KG: α-ketoglutarate; GSH: reduced glutathione; GSSG: oxidized glutathione; PPP: pentose phosphate pathway; 1C metabolism: one-carbon metabolism; PUFA: polyunsaturated fatty acid; PUFAOOH: polyunsaturated fatty acid hydroperoxide; PUFA-OH: polyunsaturated fatty acid alcohol; PHGDH: phosphoglycerate dehydrogenase; PSAT1: phosphoserine aminotransferase 1; PSPH: phosphoserine phosphatase; SHMT2: serine hydroxymethyltransferase 2; TALDO1: transaldolase 1; HMOX1 (HO-1): heme oxygenase 1; TRX1: thioredoxin; TXNRD1: thioredoxin reductase 1; GPX4: glutathione peroxidase 4; GR: glutathione reductase; SLC7A11: cystine/glutamate antiporter; SLC3A2: transporter heavy chain; ROS: reactive oxygen species; Fe^2+^: ferrous iron; CO: carbon monoxide; NADPH/NADP^+^: nicotinamide adenine dinucleotide phosphate (reduced/oxidized).

Among the upregulated programs, NRF2-dependent transcriptional pathways emerged as a unifying key feature of sorafenib resistance. NRF2 functions as a master regulator of cellular redox homeostasis and metabolic adaptation, coordinating the expression of genes involved in antioxidant defense, detoxification and intermediary metabolism [22,33]. In resistant cells, we observed the induction of canonical NRF2 target genes, including *Gpx4*, *Txn1*, *Txnrd1*, *Hmox1*, *Fth1*, *Taldo1*, *Phgdh*, and *MafG*, indicating sustained activation of this regulatory axis. Importantly, acute sorafenib treatment rapidly induced a similar NRF2-dependent transcriptional response in sensitive hepatoma cells, suggesting that sorafenib resistance are initiated rapidly upon drug exposure and subsequently become stably established upon sustained Nrf2 mRNA and protein upregulation, as observed in Hep55-RES cells. Figure 9 schematically summarizes such evidences.

Although this study does not rule out the presence of additional resistance mechanisms [12], it establishes a highly reproducible model for mechanistic and translational investigations of NRF2-mediated drug resistance. Despite being derived from the single parental Hep55.1C cell line, the observation that six independently generated resistant sublines [26] converged on the same mechanism strongly suggests that this pathway represents a critical mode of resistance.

Mechanistically, the induction of NRF2 target genes supports multiple complementary antioxidant and metabolic modules. Upregulation of GPX4 and components of the glutathione system enhances detoxification of lipid peroxides, thereby limiting lipid peroxidation and reducing susceptibility to ferroptosis [37,38]. Concurrent activation of the thioredoxin system provides an additional NADPH-dependent antioxidant axis essential for maintaining protein redox balance under oxidative stress [20]. Sustained oxidative stress is known to increase cancer cell dependence on the thioredoxin axis to preserve redox equilibrium and survival [39]. In parallel, induction of the heme detoxification pathway through *Hmox1* and ferritin subunits limits iron-mediated reactive oxygen species generation. Together, these modules constitute an antioxidant network capable of buffering sorafenib-induced oxidative damage. A study providing an in-depth investigation of the role of Hmox1 in the development of sorafenib resistance in Hep55.1C cells has been accepted for publication [40].

Maintenance of these antioxidant defenses requires continuous replenishment of the reducing equivalents GSH and NADPH. Consistent with this requirement, resistant cells exhibited increased expression of metabolic enzymes supporting NADPH and glutathione homeostasis, including transaldolase (*Taldo1*) and phosphoglycerate dehydrogenase (*Phgdh*), both induced by NRF2. Although these enzymes do not directly generate NADPH, they promote metabolic flux through the pentose phosphate pathway and serine/one-carbon metabolism, thereby indirectly sustaining reducing capacity [21,41,42]. For example, though transaldolase does not produce directly NADPH, it allows pentose phosphates to be converted back into glycolytic intermediates (e.g., fructose-6-phosphate), which can then reenter into the oxidative PPP, essential to maintain NADPH levels. Analogously, PHGDH does not produce NADPH directly, but it enhances NADPH generation indirectly via the serine/one-carbon metabolism. Furthermore, PHGDH supports the production of glutathione via the methionine cycle. PHGDH overexpression, as well as other genes involved in the serine synthesis pathways like PSAT1 and SHMT2, are induced by NRF2 to support redox homeostasis, NADPH production and resistance to oxidative stress [43]. PHGDH itself was shown to promote sorafenib resistance [16]. These findings are consistent with accumulating evidence linking NRF2 activation to metabolic rewiring that supports redox balance and therapeutic resistance.

MafG (small musculoaponeurotic fibrosarcoma oncogene homolog G), a member of the small Maf protein family, forms a heterodimer with NRF2 to generate the transcriptionally active NRF2–MafG complex, which binds to Antioxidant Response Elements (AREs) within the promoters of cytoprotective genes. The observed upregulation of MafG itself further reinforces the NRF2-driven transcriptional network. [44].

Taken together, these observations indicate that NRF2-driven transcriptional reprogramming constitutes a central adaptive mechanism conferring resistance to sorafenib. This phenotype is characterized by a coordinated antioxidant response that attenuates oxidative stress, suppresses ferroptosis and promotes metabolic flexibility under pharmacologic stress. In established tumors, constitutive NRF2 activation has been extensively linked to resistance against multiple chemotherapeutic agents, including cisplatin, doxorubicin, paclitaxel, as well as to radioresistance [22,23,24,25]. The present findings extend this paradigm to targeted therapies, demonstrating that NRF2-mediated adaptive programs can also weaken the efficacy of multikinase inhibitors. This highlights the possibility that NRF2 activation may represent a mechanism of acquired resistance across multiple therapeutic approaches in HCC and potentially other malignancies.

Functional involvement of NRF2-dependent pathways was supported by pharmacological inhibition using brusatol. While brusatol does not directly inhibit NRF2 transcriptional activity, its suppression of protein synthesis preferentially affects short-lived proteins such as NRF2 and its downstream effectors. In sorafenib-resistant cells, brusatol markedly reduced cell viability and restored sensitivity to sorafenib. Notably, loss of viability correlated with depletion of antioxidant proteins rather than with transcriptional changes, underscoring the importance of post-transcriptional regulation in maintaining resistance.

Some limitations of this work should be acknowledged. First, while pharmacological inhibition provides functional support, genetic approaches targeting NRF2 would further strengthen causal inference. Second, although multiple components of ferroptosis-regulatory pathways were induced, ferroptotic cell death was not directly measured; therefore, conclusions regarding ferroptosis suppression are based on pathway-level inference. Addressing these aspects in future studies will further refine the mechanistic understanding of sorafenib resistance. Finally, while our model is murine, the molecular principles uncovered are highly conserved and provide mechanistic insight into NRF2-mediated drug resistance that is likely relevant to human disease.

Despite these limitations, our findings are consistent with a growing body of literature identifying NRF2 activation as a general mechanism of resistance to chemotherapy and radiotherapy [22,23,24,25]. In hepatocellular carcinoma, where oxidative stress is a key determinant of therapeutic response, NRF2-mediated redox adaptation may represent a particularly critical survival strategy. Importantly, several components of the NRF2-regulated network identified here, including GPX4, the thioredoxin system, and PHGDH-driven metabolic pathways, are emerging as pharmacologically targetable vulnerabilities [16,20,45,46,47,48,49,50,51,52,53,54,55,56,57,58].

From a translational perspective, these results support the rationale for combination strategies aimed at disrupting redox homeostasis to overcome sorafenib resistance. While direct NRF2 inhibition remains challenging due to specificity and toxicity concerns, targeting downstream antioxidant and metabolic dependencies may offer better therapeutic opportunities. Rational combinations that impair redox buffering capacity could lower the threshold for sorafenib-induced oxidative damage and restore tumor cell susceptibility. Toward this aim, a manuscript on Hmox1 inhibition is in press [40] and another addressing Phgdh targeting is currently in preparation. Additional work is ongoing to investigate Gpx4 and the Trx/TrxR systems.

In conclusion, this study demonstrates that acquired resistance to sorafenib in this model of hepatocellular carcinoma is associated with a stable NRF2-dependent transcriptional and metabolic reprogramming that enhances antioxidant defenses, supports metabolic flexibility and promotes tumor cell survival under therapeutic stress. Through the coordinated activation of redox-regulatory pathways, including glutathione- and thioredoxin-based antioxidant systems, heme detoxification and NADPH-supporting metabolic circuits, resistant cells effectively buffer sorafenib-induced oxidative damage and evade regulated cell death programs.

By integrating transcriptomic profiling with functional pharmacological inhibition, our findings identify NRF2-regulated redox metabolism as a central adaptive mechanism underlying sorafenib resistance. These results provide mechanistic insights into the adaptive plasticity of hepatocellular carcinoma and highlight redox homeostasis as a critical therapeutic vulnerability. Targeting NRF2-dependent antioxidant and metabolic pathways may therefore represent a promising strategy to restore sorafenib sensitivity and improve therapeutic outcomes in patients with advanced HCC.

## Figures and Tables

**Table 1:** Primers employed in ddPCR experiments.

Gene	Species	Forward Primer	Reverse Primer
*Actb (β-actin)*	Mus musculus	CAGCCTTCCTTCTTGGGTATG	GGCATAGAGGTCTTTACGGATG
*Phgdh*	Mus musculus	CCTTAGTGGACCACGAGAATG	CACAAACTGGACTGCGATTTC
*Hmox1*	Mus musculus	CTCCCTGTGTTTCCTTTCTCTC	GCTGCTGGTTTCAAAGTTCAG
*Nfe2l2 (NRF2)*	Mus musculus	CTCCGTGGAGTCTTCCATTTAC	GCACTATCTAGCTCCTCCATTTC
*Txn1*	Mus musculus	GTCTATACCCAACTGCCATCTG	GGGACTAAATCAGGCCATTTCT
*Txnrd1*	Mus musculus	AAGTGGGTGAGATGGCTTATG	GGAGACAATGCTACACCAGTTA
*Fth1*	Mus musculus	TGTATGCCTCCTACGTCTATCT	CCTCATGAGATTGGTGGAGAAA
*Taldo1*	Mus musculus	CTGCACAACGAAGACCAAATG	GAGCATCCGCTCCAACTTTA
*Gpx4*	Mus musculus	CCGATATGCTGAGTGTGGTTTA	GGCTGCAAACTCCTTGATTTC

## Data Availability

The authors declare that all the data supporting the findings of this study are available within the article and its Supplementary Information Files. The RNA-seq raw data generated and analyzed during the current study are available in the NCBI Gene Expression Omnibus (GEO) data repository with the accession number: (provided upon acceptance for publication).

## Supplementary Materials

The following supporting information can be downloaded at: https://www.mdpi.com/article/10.3390/biotech15010018/s1. Supplementary Table S1. Gene Set Enrichment Analysis (GSEA). Molecular pathways significantly enriched for overexpressed genes in sorafenib-resistant cells.
